# A pelvic kinematic approach for calculating hip angles for active hip disarticulation prosthesis control

**DOI:** 10.1186/s12984-023-01273-x

**Published:** 2023-11-09

**Authors:** Farshad Golshan, Natalie Baddour, Hossein Gholizadeh, Edward D. Lemaire

**Affiliations:** 1 Faculty of Engineering, Department of Mechanical Engineering, University of Ottawa, Ottawa, Canada; 2 Ottawa Hospital Research Institute, Centre for Rehabilitation Research and Development, Ottawa, Canada

**Keywords:** Gait analysis, Hip angle calculation, Active hip prostheses, Motorized prostheses control, Pelvic motion analysis, Transfemoral prosthetic gait

## Abstract

**Background:**

Control system design for a microprocessor-controlled hip–knee–ankle–foot (HKAF) prosthesis is a challenge since hip disarticulation amputees lack the entire leg and, therefore, only have pelvis movement as user-guided input. This research proposes a method for determining hip joint angles from pelvis movement in a control system for the next generation of powered prostheses.

**Method:**

Three-dimensional pelvic motion and stance time of 10 transfemoral (TF) prosthetic users were used to identify important features and to develop an algorithm to calculate hip angles from pelvis movement based on correlation and linear regression results. The algorithm was then applied to a separate (independent) TF group to validate algorithm performance.

**Results:**

The proposed algorithm calculated viable hip angles during walking by utilizing pelvic rotation, pelvic tilt, and stance time. Small angular differences were found between the algorithm results and motion capture data. The greatest difference was for hip maximum extension angle (2.5 ± 2.0°).

**Conclusions:**

Since differences between algorithm output and motion data were within participant standard deviations, the developed algorithm could be used to determine the desired hip angle from pelvis movements. This study will aid the future development of gait control systems for new active HKAF prostheses.

**Supplementary Information:**

The online version contains supplementary material available at 10.1186/s12984-023-01273-x.

## Introduction

People with an amputation at the hip level have the most difficulty returning to walking [1]. Operating a passive hip disarticulation (HD) prosthesis can be physically demanding, especially for elderly users, and requires sufficient physical fitness [2, 3]. Furthermore, in cases where people continued to use their prostheses, excessive pelvic tilt and rotation during ambulation could eventually lead to spinal injuries [4]. The lack of muscle power at the hip, knee, and ankle/foot can also result in a fixed and slow cadence. A possible solution to reduce physical demand and improve mobility in people with HD amputation is to utilize active actuators to operate the hip joint intelligently. User input is required for hip control; however, identifying viable user input that could be used for prosthetic control is a challenge since hip disarticulation amputees lack the entire hip and leg.

In conjunction with knowledge of 3D gait biomechanics, advancements in actuator and sensor technologies have produced effective control systems for lower extremity prostheses [5]. Powered knee joints that actively aid the prosthetic user in challenging terrains can be beneficial by reducing strain on the body [6, 7]. However, microprocessor-controlled and powered hip joints are only now being investigated in the research domain [8].

Ueyama et al. [8] substituted conventional prosthetic hip and knee joints with conventional fully passive (mechanical) hip joints and semi-active (active dampening) knee joints with robotic motorized joints to actuate the knee and hip. Their prototype used a simple gait control strategy to mirror intact limb motion (echo-controller). A feedback controller allowed the hip to extend while compressing a virtual spring as the participant moved the intact limb forward. The compressed virtual spring's potential energy was released during swing phase, allowing the actuators to swing the prosthesis forward. While this gait control method produced natural gait patterns, the control method was not viable for day-to-day activities due to limited user control and overreliance on the regularity of the steady state gait [9]. Delayed response is another disadvantage of echo controllers [10]. Real-time user control becomes especially crucial while operating a powerful joint capable of propelling the user forward and delayed response of the prosthesis may lead to loss of balance [11]. Mirroring the intact limb is also problematic for stumbles or other asymmetric gait activities (e.g., working in a kitchen, etc.). With the evolution of hip prosthetic actuation, the need for a robust and reliable control mechanism has become essential [12].

Similar to transfemoral (TF), some people with HD amputation are fitted with a prosthesis and receive rehabilitation to use the new prosthesis appropriately [13]. Unlike TF amputees that utilize thigh skeletomuscular segments to help control their prosthesis, HD patients typically rely on their pelvis and torso to operate and control their passive hip–knee–ankle–foot (HKAF) prosthesis [14–16]. Since motorized HD prosthesis kinetics and kinematics differ from gait with passive devices [8], applied torque through the motorized hip joint will require a specific gait control strategy [12]. That is, conventional mechanical HD prosthesis biomechanics cannot be directly used for optimal motorized hip joint performance. A more appropriate comparator for walking with a powered hip joint would be TF amputee gait since both powered-hip-HKAF, and TF prostheses can have similar prosthetic knee joints and feet; therefore, a well controlled hip joint could allow an HD amputee to walk as well as a TF amputee [17]. The sole body segment an HD amputee can directly control is the pelvis. Thus, understanding how TF amputees' pelvic motion relates to hip angle during the gait cycle can be the basis of a powered hip joint control approach.

In this research, we identified common pelvic motion features during the gait cycle among TF prosthesis users and determined how these features influence hip flexion and extension. These pelvic features and their relationship with hip rotation were then used to develop an algorithm to estimate the hip angle at each instance in the gait cycle. Thus, this algorithm enables hip angle to be calculated from pelvic motion measurements. Successful evaluation of this algorithm with a separate TF amputee gait dataset demonstrates the viability of this new approach. This pelvis-based approach enables progression to the next phase of powered hip joint control system development, leading to better mobility for people with hip level amputations.

## Methods

We investigated TF amputee pelvic motions (pelvic tilt, pelvic obliquity, pelvic rotation) and their relationship with hip rotation. This data was used to develop an algorithm to calculate hip angles throughout the gait cycle, from pelvic motion while walking on a treadmill with no incline. Hip angles calculated by the algorithm were compared with measured (motion-captured) hip angles to quantify algorithm. This study focused on analyzing model performance during steady-state walking.

### Databases

Two databases of transfemoral amputee gait were used in this study, for algorithm development and validation. Dataset selection requirements were: all participants must be TF amputees; participants must ambulate without relying on walking assistive devices such as canes, walkers, or treadmill assistive bars; gait data must be from steady-state sequences of level walking tests.

A database from a group of 10 people with unilateral transfemoral amputation with K3 and K4 activity levels [18] was used for algorithm development. Data were collected in the CAREN Extended virtual reality laboratory at The Ottawa Hospital Rehabilitation Centre and processed with C-motion Visual 3D (version 6) using six degrees of freedom model and MATLAB software version 2021a [19–22]. People walked on a treadmill (level walking) at self-selected walking speeds (1.05 ± 0.27m/s), and ten strides per person were extracted (a total of 100 strides). The development group's mean age was 47 (± 9.4) years, weight was 85 (± 8.6) kg, and height was 176 (± 9) cm. The gait events were processed using foot velocity relative to the pelvis method [23] and through manual verification. This database has been utilized in various biomechanics-related studies published in literature [21, 24, 25]. The Ottawa Health Science Network Research Ethics Board approved the secondary use of this dataset.

Testing group data were obtained from a recently published database ([26, 27]) that used 10 VICON 3D motion capture cameras and a dual belt instrumented Bertec treadmill. Ten people with unilateral transfemoral amputation with K2 and K3 activity levels met our inclusion criteria. Participants walked at different fixed speeds based on their activity level. People with K2 activity levels completed separate walking trials at 0.4, 0.5, 0.6, 0.7, and 0.8 m/s. Those with K3 activity levels walked at 0.6, 0.8, 1.0, 1.2, and 1.4 m/s. The marker data were then processed using modified Vicon plug-in-gait model to obtain the hip and knee kinematics [28, 29]. The pelvic kinematic data were processed using a CODA pelvic model in MATLAB™ R2021a [30]. The testing group's mean age was 47 (± 15) years, weight was 84.5 (± 17.6) kg, and height was 177 (± 11) cm.

Differences between the development and testing groups for activity level, walking speed, or prosthetic components are desirable when evaluating model performance on different groups of participants. This helps to determine model generalizability and avoid cases where better results are due to training and testing on the same group. Prosthesis details for both groups are presented in Tables 1 and 2.

**Table 1:** Testing group details

PNO	Mobility class	Prosthetic knee	Knee control method	Number of strides
1	K3	Plié^1^	Microprocessor	166
2	K2	C-leg^2^	Microprocessor	263
3	K3	C-leg^2^	Microprocessor	232
4	K3	Rheo^3^	Microprocessor	210
5	K2	C-leg^2^	Microprocessor	249
6	K3	C-leg^2^	Microprocessor	243
7	K2	C-leg^2^	Microprocessor	235
8	K3	C-leg^2^	Microprocessor	310
9	K3	Plié^1^	Microprocessor	285
10	K3	C-leg^2^	Microprocessor	210

Manufacturers: 1 = Freedom innovations, 2 = Ottobock, 3 = Össur

**Table 2:** Development group details

PNO	Mobility Class	Prosthetic knee	Knee control method	Number of strides
1	K4	C-leg^1^	Microprocessor	10
2	K4	C-leg^1^	Microprocessor	10
3	K3	C-leg^1^	Microprocessor	10
4	K3	C-leg^1^	Microprocessor	10
5	K4	Endolite	Mechanical	10
6	K4	Mauch^2^	Mechanical	10
7	K3	C-leg^1^	Microprocessor	10
8	K4	Trulife	Mechanical	10
9	K3	X3^1^	Microprocessor	10
10	K4	X3^1^	Microprocessor	10

Manufacturers: 1 = Ottobock, 2 = Össur

### Algorithm development

Pelvic movement in sagittal (anterior/posterior tilt), frontal (lateral tilt or obliquity), and transverse (pelvic rotation) planes were investigated, and gait phase transition timing was assessed to determine their correlation with hip angle throughout the gait cycle.

In this study, any posterior movement of the pelvis and hip joints were considered as positive angular displacement (posterior pelvic tilt, forward pelvic rotation, and hip flexion), while anterior movements were considered as negative angular displacement (anterior pelvic tilt, backward pelvic rotation, and hip extension).

The hip angle calculation algorithm development required:

A. Hip angle features: Temporal and spatial hip angle features to calculate hip angular velocity.
B. Correlations: Analyze pelvic motion and stance time data to determine common features and correlations with hip angle features.
C. Hip angle feature calculation equations: Develop regression equations to calculate the hip angle features using the identified pelvic features in step B.
D. Identification of per-person constants: Determine constant parameters unique to each person's gait characteristics that could be used for algorithm development.
E. Sequential hip angle calculation: Develop an algorithm for real-time hip angle calculation using parameters obtained in steps C and D.
F. Algorithm performance analysis: Evaluate the model with a new data set (testing group).

Each of these will be considered in turn.

A. Hip angle features

A typical gait cycle for people with transfemoral amputation includes ([17, 31]): foot contacts the ground (foot strike) with the hip flexed to ~ 30°; hip moves to ~ 5° of extension at the end of stance; hip starts to flex ~ 35° until 80%-90% of stride; and hip flexion decreases ~ 5° in terminal swing to ensure that the prosthetic knee is fully extended before the next foot-strike [32–35].

**Fig. 1:**
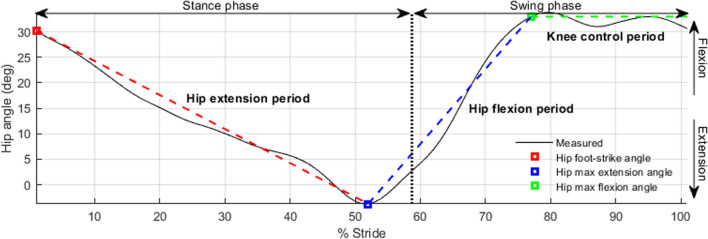
Three hip angle periods during gait cycle: hip rotation toward negative angle (extension), hip rotation toward positive angle (flexion), constant angle. Measured curve is the average of 100 strides in the development group

Hip angle kinematics can be divided into three periods (Fig. 1), with each period having a linear progression of hip angle by time (i.e., constant slope or constant angular velocity).

- Period 1 (hip extension): Initiates at foot-strike and ends at 50–60% of the gait cycle. The angular velocity vector is always negative.
- Period 2 (hip flexion): Initiates at hip max extension and continues through the stance-to-swing transition with minor angular velocity change. The velocity vector is always positive.
- Period 3 (knee control): Knee joint behavior directly affects hip rotation in this period, with hip rotation acting to keep the prosthetic knee fully extended. For this study, hip angle throughout period 3 was assumed to be constant (angular velocity is zero) until the next foot strike.

Hip angular velocity was assumed to remain mostly constant throughout each period, simplifying the hip angle calculation. Five features were present for all participants; therefore, these features were used to calculate the constant angular velocity during each period. Spatial features were hip angle at foot strike (Hθ_FS_), maximum hip extension angle (Hθ_E_), and maximum hip flexion angle (Hθ_F_). Temporal features were maximum hip extension time (Hτ_E_) and maximum hip flexion time (Hτ_F_).

For period 1 (hip extension), the constant angular velocity was calculated using Hθ_FS_, Hθ_E_, and the difference in time between the two features. For period 2 (hip flexion), the constant angular velocity was calculated using Hθ_E_, Hθ_F_, and the difference in time between the two features. In period 3 (knee control), hip angle was assumed to be constant at Hθ_F_ (i.e., zero angular velocity).

The algorithm uses these angular velocity values to calculate hip angle at each time point. Therefore, equations that define the relationships between pelvic kinematics and hip features are required.

B. Correlations

**Fig. 2:**
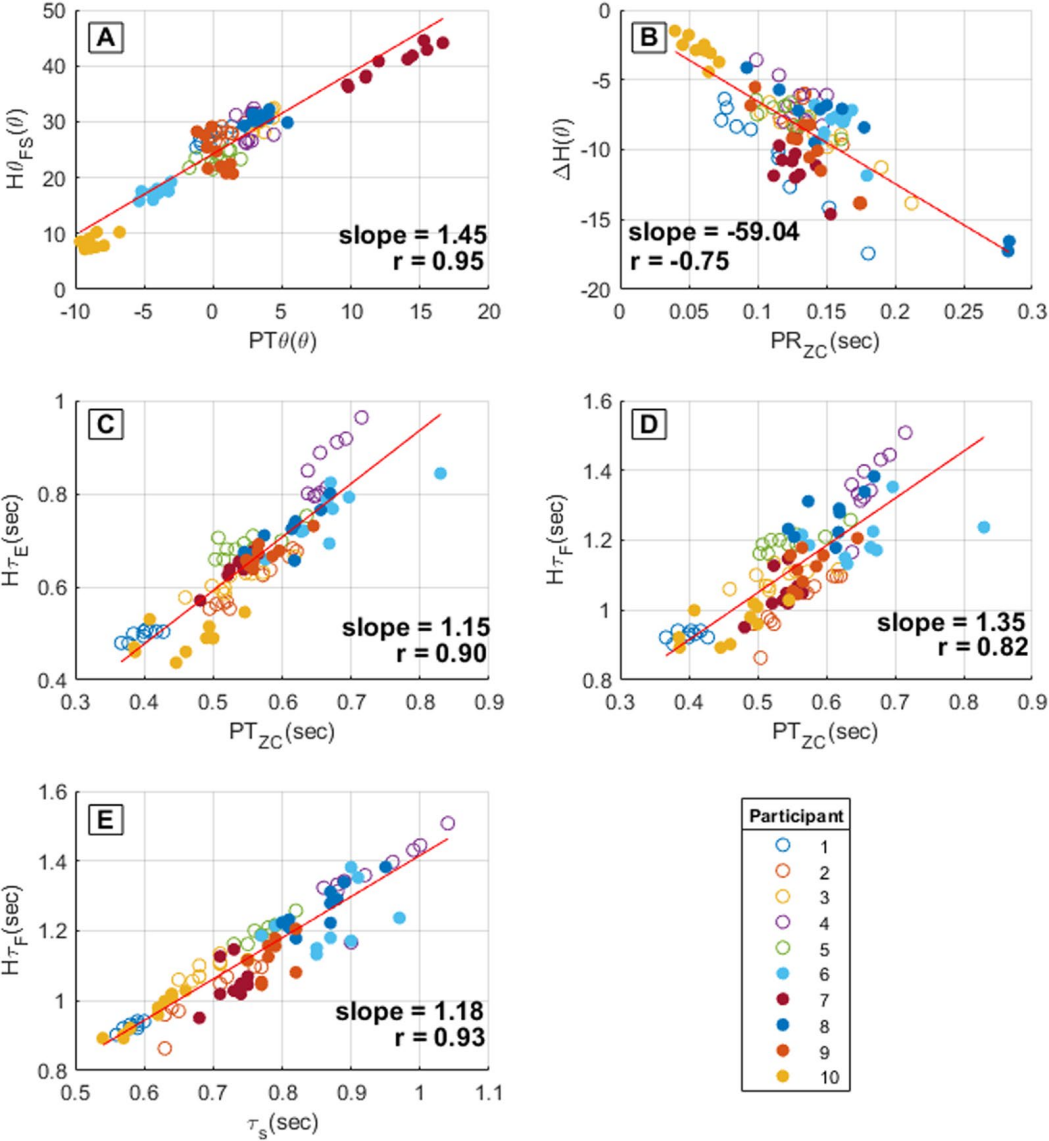
Pelvic feature and stance time correlation with hip angle features. **A** Pelvic tilt angle at foot strike (PTθ) and hip angle at foot-strike (Hθ_FS_), **B** timing of pelvic angular velocity zero-crossing in early stance (PR_ZC_) and hip angle range of motion during that period (ΔH), **C** timing of pelvic tilt angular velocity first zero crossing in midstance (PT_ZC_) and hip max extension time (Hτ_E_), **D** timing of pelvic tilt angular velocity first zero crossing in mid-swing (PT_ZC_) and hip max flexion time (Hτ_F_), **E** stance time (τs) and hip max flexion time (Hτ_F_)

Pelvic angular velocity, pelvic tilt, pelvic obliquity, pelvic rotation, and stance time per stride for each person in the development group were analyzed to determine common features that could be used to calculate the constant hip angular displacement and velocities. Twenty-two features were identified as potential candidates. Next, p-earson correlation analyses were applied to each feature candidate to determine which features were most related to the hip angle features. The strongest correlations were (Fig. 2):

- Pelvic tilt angle at foot strike (PTθ) and hip angle at foot-strike (Hθ_FS_): r = 0.95
- Timing of pelvic rotation angular velocity zero-crossing in early stance (PR_ZC_) and the hip rotation angle range of motion during that period (ΔH): r = -0.75
- Timing of pelvic tilt angular velocity first zero crossing in midstance (PT_ZC_) and hip max extension time (Hτ_E_): r = 0.90
- PT_ZC_ and hip max flexion time (Hτ_F_): r = 0.82
- Stance time (τs) and hip max flexion time (Hτ_F_): r = 0.93.

**Fig. 3:**
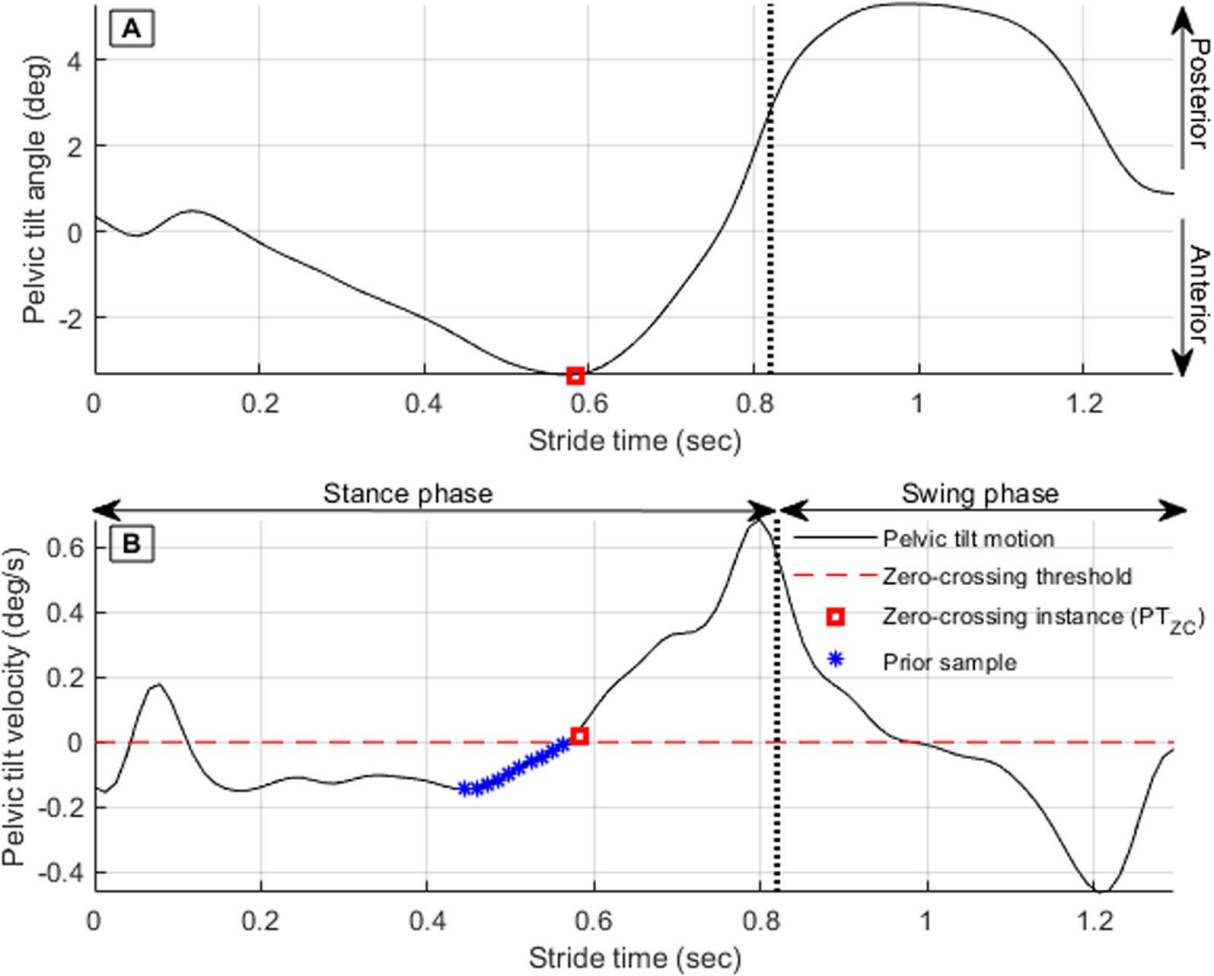
Pelvic tilt angular displacement (**A**) and corresponding pelvic tilt angular velocity (**B**). The red square represents the most recent sample, while the blue stars represent the 10 data samples prior to the most recent sample

PT_ZC_ (Timing of pelvic rotation angular velocity zero-crossing in early stance) can be detected in real-time by continuously monitoring the 10 most recent pelvic tilt angular velocity data samples. If the most recent sample is positive while the prior 10 samples are negative, positive zero-crossing is detected (Fig. 3).

**Fig. 4:**
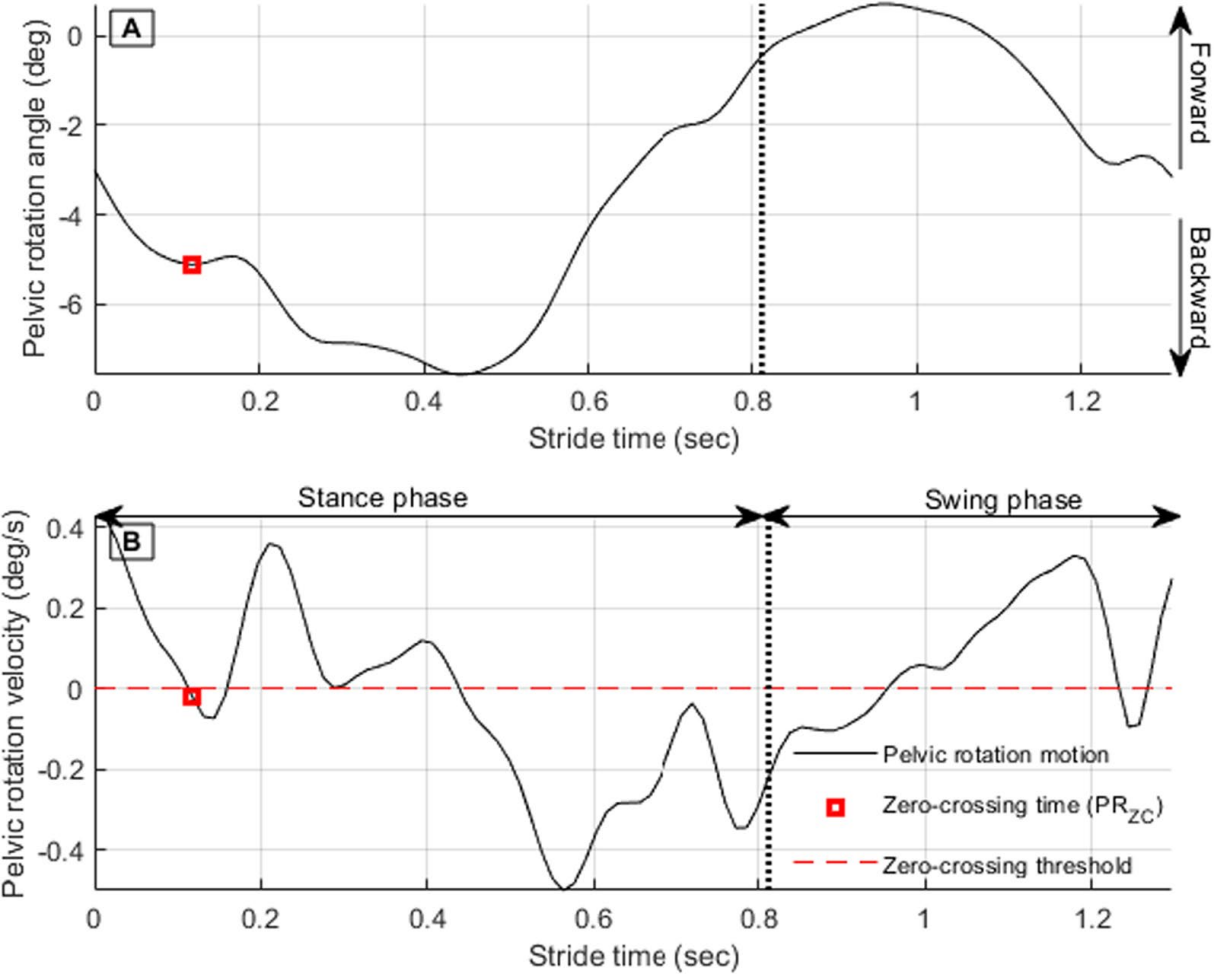
Pelvic rotation angular displacement (**A**) and corresponding pelvic rotation angular velocity (**B**)

PR_ZC_ (timing of pelvic tilt angular velocity first zero crossing in mid-swing) is detectable by measuring the pelvic rotation angular velocity zero-crossing (Fig. 4). Initially, angular velocity should be in the positive region. The crossing instance is marked if the angular velocity vector crosses the zero value into the negative angular velocity region.

C. Hip angle feature calculation equations

A series of linear regression equations were determined to calculate the hip angle features based on the linear relationships and correlations between pelvic motion features, gait events, and hip rotation features (Fig. 2). These regression equations are calculated sequentially (from Eqs. 1–5) as the pelvic features and gait events are detected (Fig. 6).

When foot strike is detected, regression Eq. 1 is used to calculate hip angle.

1 $${H\theta }_{FS}=24.27+\left(1.45\times PT\theta \right)$$

where Hθ_FS_ is hip angle at foot strike and PTθ is pelvic tilt angle at foot strike.

Once pelvic rotation zero crossing is detected (Fig. 4), regression Eq. 2 is used to determines ΔH.

2 $$\Delta H=-0.6261-\left(59.04\times {PR}_{ZC}\right)$$

where PR_ZC_ is the difference between the time of pelvic rotation angular velocity first zero crossing and foot-strike time. ΔH is the difference between hip angle at foot-strike and hip angle at PR_ZC_ time.

Approaching the end of stance phase, pelvic tilt zero crossing is detected (Fig. 3) and used to calculate hip max extension time using regression Eq. 3 and hip max flexion time using Eq. 4.

3 $${H\uptau }_{E}={0.0462+(1.15\times PT}_{ZC})$$

4 $${H\uptau }_{F\alpha }=0.3461 +\left(1.35\times {PT}_{ZC}\right)$$

where PT_ZC_ is the time of pelvic tilt angular velocity zero crossing, Hτ_E_ is the hip max extension time, and Hτ_Fα_ is the hip max flexion time at the PT_ZC_ instant.

During the foot-off instance, Hτ_F_ is recalculated again in Eq. 5 to take advantage of the higher Hτ_F_ and τ_S_ correlation value (R = 0.94) compared to Hτ_F_ and PT_ZC_ (R = 0.84).

5 $${H\uptau }_{F\beta }=0.0874 +\left(1.18\times {\uptau }_{S}\right)$$

where τ_S_ is the gait stance time, and Hτ_Fβ_ is max hip flexion time at foot-off.

D. Per person, unique constants

For hip angle calculation using the developed algorithm, two unique input constants were defined for each person after analyzing the development dataset: mean hip max extension and mean hip max flexion angles. In practice, during the fitting process, a prosthetist would increase or decrease these constants for each patient until results are satisfactory [36]. That is, iterative changes are made to the values during the prosthetic fitting process until gait is acceptable to the end-user and clinician, similar to the process for tuning other microprocessor controlled prosthetic joints.

Hip max extension and max flexion angles tended to be in a unique but limited range for each TF amputee in the development group. Studies suggested that this unique hip ROM is due to prosthesis user control strategies to maintain balance during walking [37, 38]. The per-person-specific hip max extension and max flexion angle constants for both development and testing groups are provided in Table 3.

**Table 3:** Per-person unique constants of development and testing group datasets

PNO	Development group		Testing group	
	${{\overline{\text{H}\theta}}_{\text{E}}}$	${{\overline{\text{H}\theta}}_{\text{F}}}$	${{\overline{\text{H}\theta}}_{\text{E}}}$	${{\overline{\text{H}\theta}}_{\text{F}}}$
1	− 23.22	27.86	− 4.69	37.14
2	− 17.55	24.06	− 5.1	27.93
3	− 3.85	34.74	− 10.26	28.02
4	− 10.24	24.29	− 9.25	37.33
5	− 22.56	28.52	− 10.88	32.82
6	− 25.02	16.05	− 7.61	41.01
7	− 12.74	33.50	− 4.64	27.35
8	− 1.55	36.81	− 9.52	32.64
9	− 17.80	26.15	− 7.16	32.03
10	− 20.22	7.54	− 15.94	38.64

${{\overline{\text{H}\theta}}_{\text{E}}}$: Hip max extension angle constant, ${{\overline{\text{H}\theta}}_{\text{F}}}$: Hip max flexion angle constant

E. Hip angle calculation

**Fig. 5:**
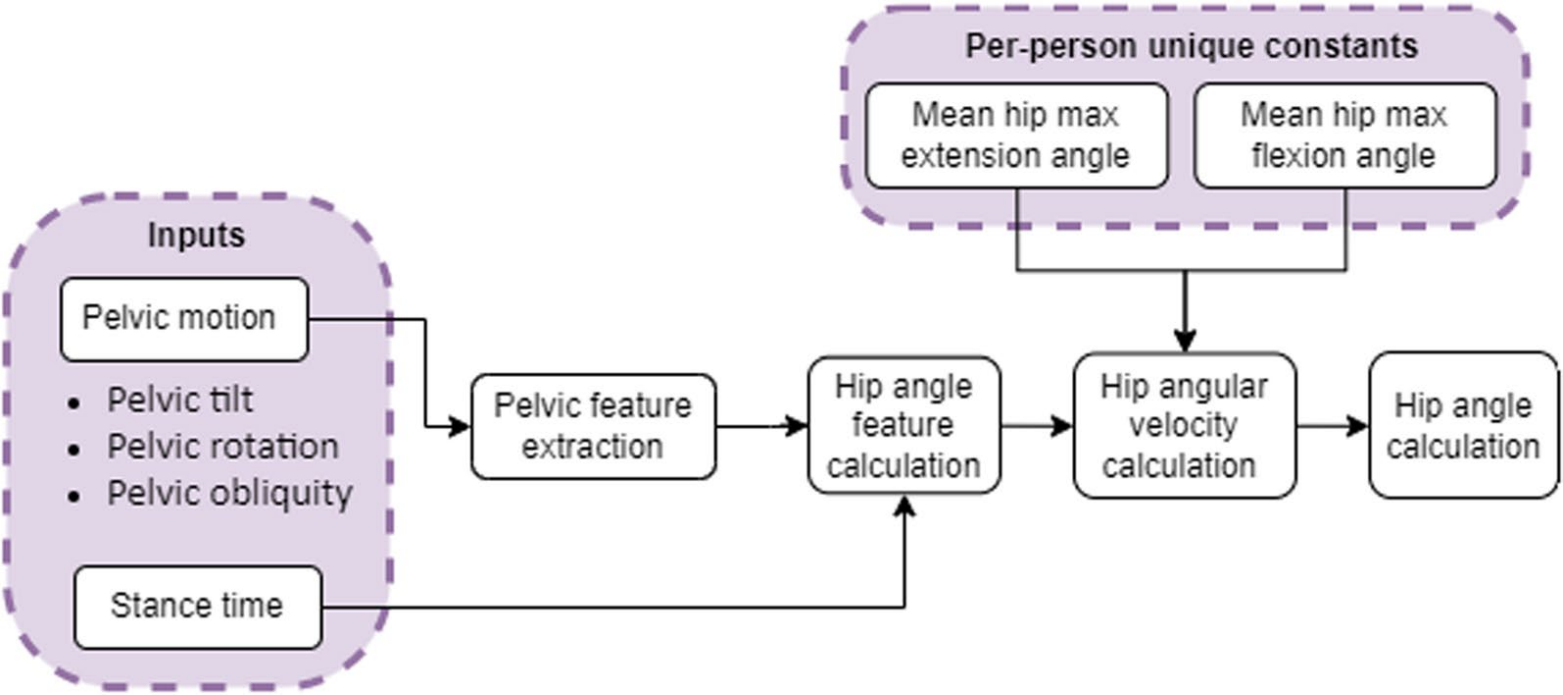
Gait cycle hip angle calculation block diagram

Figure 5 shows the general block diagram of the hip angle calculation algorithm for steady state gait. Initially, pelvic features are extracted from pelvic motion data. Then, using the hip angle feature calculation equations and foot-off time, the hip parameters necessary for constant hip angular velocity calculations are obtained. In the algorithm's last two stages, the calculated hip features are used to calculate hip angular velocity and hip angle.

**Fig. 6:**
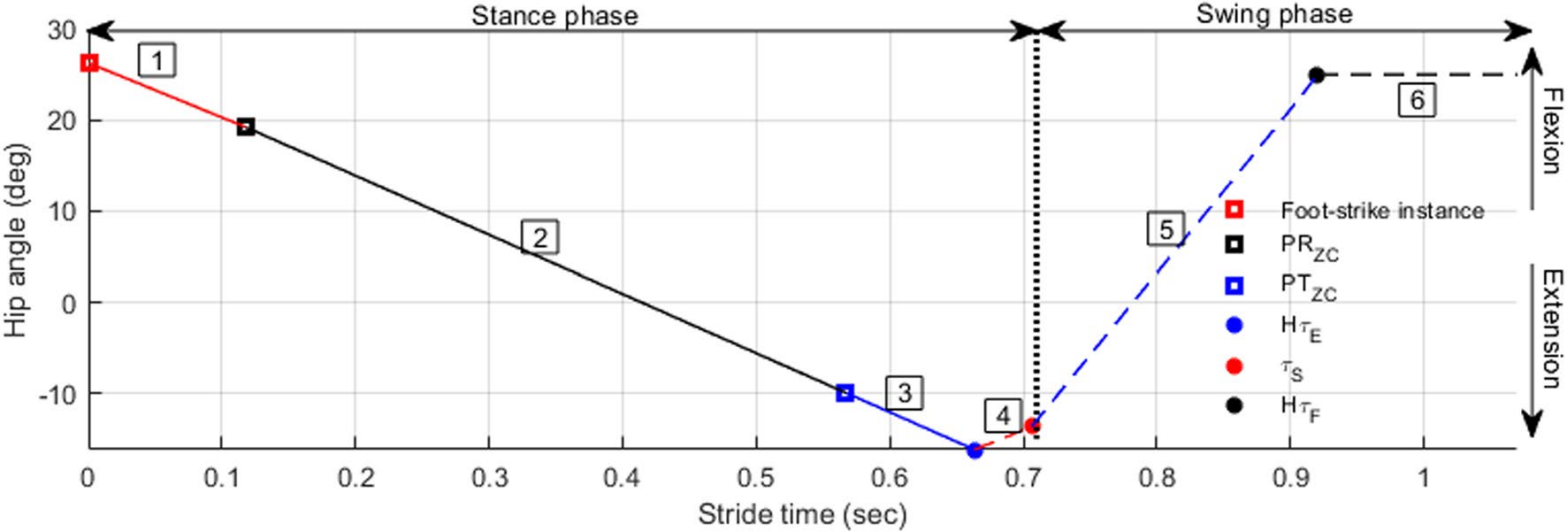
Calculated hip angle throughout the gait cycle. PR_ZC_: pelvic rotation zero-crossing time, PT_ZC_: pelvic tilt zero-crossing time, Hτ_E_: hip max extension time, τ_s_: stance time, Hτ_F_: hip max flexion time. Sequences are foot-strike to PR_ZC_ (sequence 1, solid red line), PR_ZC_ to PT_ZC_ (sequence 2, solid black line), PT_ZC_ to Hτ_E_ (sequence 3, solid blue line), Hτ_E_ to τ_FO_ (sequence 4, red dashed line), τ_FO_ to Hτ_F_ (sequence 5, blue dashed line), and Hτ_F_ to the end of the gait cycle (sequence 6, black dashed line)

The six sequences shown in Fig. 6 represent five different hip angle equations used in the algorithm. Since the algorithm was developed to run in real-time, pelvic features are detected by the algorithm at different times during the gait cycle (since people walk at different speeds). Therefore, different hip calculation equations are used depending on the timing of each pelvic feature detection. The hip extension angle (period 1) is calculated during the first three sequences. During sequences 4 and 5, the hip angle during hip flexion (period 2) is calculated, and during sequence 6 the hip angle during period 3 is calculated. For detailed mathematics of hip calculations for each sequence see Additional file 1.

F. Algorithm evaluation

The developed algorithm was applied to both testing and development groups. Algorithm performance was assessed by calculating hip angle feature differences between calculated (algorithm) and motion capture system data (ground truth). To provide a reference for whether the differences were appropriate or beyond typical participant variability, the calculated differences were compared with participant averaged hip feature standard deviations in the testing and development set.

## Results

### Development group

The root-mean-square-error (RMSE) between the measured and calculated hip trajectory and the average differences between motion-captured features and algorithm calculated data for the development group are shown in Table 4. Participants walked at self-paced speeds ranging from 0.67 m/s to 1.55 m/s, with a mean of 1.05 ± 0.27 m/s.

**Table 4:** Development group average differences and standard deviations between measured and calculated features for all participants, average correlation between calculated and measured features, and average root-mean-squared-error between calculated and measured hip trajectories

PNO	Walking speed (m/s)	Hθ_FS_	Hθ_E_	Hτ_E_	Hθ_F_	Hτ_F_	Corr	RMSE
1	1.50 ± 0.01	3.0 ± 1.0	1.9 ± 1.6	0.0 ± 0.0	1.1 ± 1.2	0.2 ± 0.0	0.98 ± 0.01	3.26 ± 0.73
2	1.23 ± 0.16	2.5 ± 0.9	1.7 ± 1.1	0.0 ± 0.0	2.8 ± 1.5	0.1 ± 0.0	0.99 ± 0.00	3.40 ± 0.77
3	0.91 ± 0.06	1.1 ± 1.0	2.6 ± 1.8	0.0 ± 0.0	1.0 ± 1.4	0.2 ± 0.0	0.94 ± 0.03	2.11 ± 0.57
4	0.76 ± 0.18	2.6 ± 2.8	3.3 ± 1.9	0.1 ± 0.0	1.8 ± 1.9	0.2 ± 0.1	0.96 ± 0.02	4.08 ± 0.74
5	1.09 ± 0.09	1.6 ± 1.4	3.5 ± 3.5	0.0 ± 0.0	2.8 ± 0.8	0.2 ± 0.0	0.98 ± 0.01	5.18 ± 1.05
6	1.00 ± 0.10	1.0 ± 0.8	3.2 ± 2.8	0.0 ± 0.0	1.9 ± 2.2	0.1 ± 0.1	0.97 ± 0.01	3.11 ± 0.56
7	1.05 ± 0.03	2.6 ± 1.1	2.5 ± 2.5	0.0 ± 0.0	3.3 ± 4.8	0.1 ± 0.1	0.95 ± 0.03	4.06 ± 0.46
8	0.67 ± 0.06	1.7 ± 1.4	1.8 ± 2.1	0.0 ± 0.0	3.1 ± 2.0	0.2 ± 0.0	0.99 ± 0.01	4.09 ± 0.82
9	0.93 ± 0.13	3.6 ± 4.2	2.3 ± 2.6	0.0 ± 0.0	1.7 ± 2.1	0.1 ± 0.0	0.96 ± 0.02	3.13 ± 0.55
10	1.31 ± 0.17	3.2 ± 1.2	1.5 ± 1.1	0.1 ± 0.1	1.6 ± 1.1	0.2 ± 0.0	0.97 ± 0.01	3.42 ± 0.85
Mean	1.05 ± 0.27	2.3 ± 1.3	2.4 ± 1.8	0.0 ± 0.0	2.1 ± 1.7	0.2 ± 0.1	0.99 ± 0.01	3.71 ± 1.13
Correlation	–	0.95	0.96	0.90	0.94	0.86	–	–

Corr shows the average correlation for each person across features

Walking speed is average and standard deviation over 10 strides, PNO.: Participant number, Hθ_FS_: Hip angle at foot-strike(°), Hθ_E_: Hip max extension angle (°), Hτ_E_: Hip max extension time (s), Hθ_F_: Hip max flexion angle(°), Hτ_F_: Hip max flexion time (s), RMSE: Root-mean-square-error of the calculated hip rotation trajectory relative to the measured hip rotation trajectory

Hip max extension angle had the largest difference compared to other features, with the per-participant averaged difference ranging from 1.5° to 3.5°. The greatest overall mean difference was for hip max extension angle (2.4 ± 1.8°). Hip temporal features (Hτ_E_ and Hτ_F_) had the lowest difference, indicating that pelvic motion temporal features were in sync with hip temporal features during the gait cycles. The calculated hip features closely agreed with the measured data, with correlation values varying from 0.94 to 0.99. Similarly, the average RMSE between the measured and calculated hip trajectory for development group was small (3.71 ± 1.13°) relative to average hip range of motion (44.05 ± 7.38°).

The mean and standard deviation of hip feature angles for each participant in the development group are provided in Table 5.

On average, standard deviations were 1.7° at foot-strike, 1.4° at max extension, and 2.0° at max flexion (Table 5). The mean differences in Table 4 were compared with the average standard deviation for each participant in Table 5. The algorithm calculated features were at most 1° greater than the average standard deviations for development group participants walking at self-paced speed.

**Table 5:** Motion captured hip angle (average and standard deviation) of development group walking at a self-paced speed

PNO	Hθ_FS_	Hθ_E_	Hθ_F_
1	27.4 ± 1.2	23.2 ± 0.6	28.7 ± 1.2
2	27.9 ± 1.1	17.6 ± 0.5	27.8 ± 1.5
3	30.2 ± 1.4	3.9 ± 0.5	34.2 ± 1.4
4	29.2 ± 2.6	12.3 ± 1.9	29.5 ± 2.4
5	23.2 ± 1.2	22.6 ± 1.3	26.1 ± 0.8
6	17.5 ± 1.0	25.0 ± 2.3	16.6 ± 2.2
7	40.4 ± 3.0	12.7 ± 3.3	34.9 ± 4.8
8	30.7 ± 0.9	1.6 ± 1.0	33.7 ± 2.0
9	24.3 ± 3.2	17.8 ± 1.5	26.7 ± 2.1
10	8.4 ± 1.1	20.2 ± 1.3	8.8 ± 1.1
Mean	25.9 ± 1.7	15.7 ± 1.4	26.7 ± 2.0

PNO: Participant number; Hθ_FS_: Hip angle at foot-strike (°), Hθ_E_: Hip max extension angle (°), Hθ_F_: Hip max flexion angle (°)

### Testing group

The algorithm was applied to 2403 strides from the testing group. The outcomes (Table 6) showed small differences between measured and algorithm calculated data. Similar to the development group results (Table 4), mean hip max extension angle had the largest difference (3.4 ± 2.8°). The average RMSE comparison between participants revealed that Participant 7 exhibited the greatest deviation from the other participants in the testing group while having the lowest correlation. All per-participant correlations were above 0.94. Per-feature correlation analysis demonstrated that the lowest correlation was for Hθ_E_ (0.79). The average RMSE of measured and algorithm calculated hip trajectories in the testing group was small (5.01 ± 2.01°) compared to the hip range of motion (43.42 ± 8.18°), but larger than the development group.

**Table 6:** Testing group average per-feature differences and standard deviations for all participants, average correlation between calculated and measured features, and average root-mean-squared-error between calculated and measured hip trajectories

PNO	Hθ_FS_	Hθ_E_	Hτ_E_	Hθ_F_	Hτ_F_	Corr	RMSE
1	2.2 ± 1.7	3.1 ± 2.2	0.1 ± 0.0	2.4 ± 1.6	0.1 ± 0.0	0.97 ± 0.01	3.94 ± 0.93
2	2.3 ± 1.4	2.8 ± 1.8	0.0 ± 0.0	2.2 ± 1.5	0.0 ± 0.1	0.96 ± 0.01	3.71 ± 0.82
3	2.2 ± 1.4	2.3 ± 1.5	0.0 ± 0.0	2.0 ± 1.1	0.2 ± 0.0	0.95 ± 0.01	5.23 ± 0.65
4	1.7 ± 1.3	3.1 ± 2.3	0.1 ± 0.0	1.9 ± 1.1	0.0 ± 0.0	0.97 ± 0.01	4.21 ± 0.95
5	1.8 ± 1.4	3.5 ± 2.4	0.1 ± 0.0	1.5 ± 1.1	0.2 ± 0.1	0.97 ± 0.02	4.52 ± 2.43
6	2.3 ± 1.7	2.9 ± 1.8	0.0 ± 0.0	2.5 ± 2.0	0.0 ± 0.1	0.94 ± 0.03	5.46 ± 1.68
7	2.8 ± 1.8	4.5 ± 4.3	0.1 ± 0.1	1.8 ± 1.4	0.3 ± 0.4	0.90 ± 0.09	7.33 ± 4.35
8	1.4 ± 1.3	2.8 ± 1.8	0.0 ± 0.0	1.4 ± 1.3	0.0 ± 0.0	0.95 ± 0.02	4.80 ± 0.99
9	1.6 ± 1.0	3.4 ± 2.6	0.0 ± 0.0	1.5 ± 1.1	0.1 ± 0.0	0.92 ± 0.02	5.89 ± 0.66
10	2.1 ± 2.1	3.1 ± 3.9	0.1 ± 0.0	2.0 ± 1.3	0.1 ± 0.0	0.98 ± 0.01	4.63 ± 0.88
Mean	2.0 ± 1.6	3.4 ± 2.8	0.1 ± 0.1	1.9 ± 1.4	0.1 ± 0.2	0.95 ± 0.04	5.01 ± 2.01
Correlation	0.88	0.79	0.95	0.91	0.91	–	–

Corr shows the average correlation for each person across features

PNO.: Participant number; Hθ_FS_: Hip angle at foot-strike (°); Hθ_E_: Hip max extension angle (°); Hτ_E_: Hip max extension time (s), Hθ_F_: Hip max flexion angle (°); Hτ_F_: Hip max flexion time (s); RMSE: Root-mean-square-error of the calculated hip rotation trajectory relative to the measured hip rotation trajectory

Measured hip features for the testing group are provided in Table 7. Although testing group participants walked at a fixed speed, the hip feature standard deviations were approximately 0.3° more than the development group results. On average, hip feature standard deviations for the testing group were 2.6° at foot-strike, 3.0° at max extension, and 1.8° at max flexion. The measured and calculated hip feature differences in Table 6 were compared with the mean hip feature standard deviations in Table 7. Algorithm-calculated hip angle at foot-strike (2.0°) and hip max flexion angle (1.9°) were within the standard deviations for each feature. However, hip max extension angle difference was overestimated by 1.2°.

**Table 7:** Motion captured hip angle (average and standard deviation) of testing group

PNO	Hθ_FS_	Hθ_E_	Hθ_F_
1	34.5 ± 3.3	4.7 ± 1.8	37.1 ± 2.9
2	23.4 ± 1.7	5.1 ± 1.9	27.9 ± 2.7
3	26.1 ± 3.1	10.3 ± 1.8	28.0 ± 2.2
4	31.3 ± 1.8	9.3 ± 2.6	37.3 ± 2.2
5	24.8 ± 2.0	10.9 ± 1.8	32.8 ± 1.9
6	29.9 ± 2.9	7.6 ± 2.2	41.0 ± 3.2
7	24.3 ± 1.8	4.6 ± 1.6	27.4 ± 2.3
8	26.7 ± 1.9	9.5 ± 2.2	32.6 ± 1.9
9	30.4 ± 2.0	7.2 ± 3.4	32.0 ± 1.8
10	34.7 ± 2.6	15.9 ± 3.0	38.6 ± 2.4
Mean	28.6 ± 2.3	8.5 ± 2.2	33.5 ± 2.3

PNO.: Participant number; Hθ_FS_: Hip angle at foot-strike (°), Hθ_E_: Hip max extension angle (°); Hθ_F_: Hip max flexion angle (°)

## Discussion

In this research, we investigated pelvic movements of transfemoral amputees and developed a viable algorithm for calculating hip angles based on pelvic motion and stance time. With the introduction of powered prosthetic joints, the limitations of existing prostheses are mitigated by transferring a large amount of the mechanical work to the actuator, reducing the reliance on the user's physical capabilities to operate the prosthesis [8, 39]. Since only the pelvis segment is available for user control of a motorized hip joint, a new approach is required to convert pelvic motion (system input) into a hip angular map for a gait controller to follow. The approach proposed in this research addresses this need.

The newly developed algorithm requires only three features (pelvic tilt angle at foot strike, timing of pelvic rotation angular velocity zero-crossing in early stance, and timing of pelvic tilt angular velocity first zero crossing in midstance) and stance time data for hip angle calculation. After pelvic angle analysis, pelvic tilt and rotation exhibited common behaviours among most people in the development and testing groups and hence could be used for hip angle calculation. Pelvic angle features that correlated well with the hip angle features were then extracted for algorithm development.

Pelvic obliquity varied greatly between strides in both groups and although some common features were found, none were viable features for a motorized hip prosthetic control system. Although the pelvic tilt zero-crossing feature (PT_ZC_) was common among all persons in both groups, pelvic tilt of participant 7 in the testing group tended to be irregular relative to other people, even though there was no gait pathology observed during data acquisition. This irregularity resulted in many pelvic feature detection errors. Adequate gait training is required to avoid pelvic motions leading to pelvic feature or hip angle errors.

Model output from both development and testing datasets had very high correlations between motion capture and algorithm calculated data (R = 0.99 for development group and R = 0.95 for testing group). The high correlations indicated that the assumed three-period hip angle model (Fig. 1) closely matched typical hip angles trends for transfemoral amputees. The low RMSE hip trajectory indicated that the calculated temporal and special features closely matched the measured data. The lowest correlation observed in the testing group results in Table 6 belonged to Hθ_E_ (0.79) and was likely due to compensatory kinematic adjustments of participants to maintain a consistent speed while walking on a fixed-speed treadmill [40].

Motion capture and algorithm-calculated hip angle features had small differences for all testing and development group participants. The largest difference was hip max extension angle for the development group (2.4 ± 1.8°) and hip max extension angle for the testing group (3.4 ± 2.8°). Furthermore, the temporal hip feature differences (hip max extension time, max flexion time) were close to zero, indicating that the algorithm can accurately calculate temporal hip features. Comparing the differences with standard deviations of both datasets showed that only the calculated hip max extension angle exceeded the participant's typical standard deviation (by 1° for development group and by 1.2° for testing group). All other hip feature differences between calculated and measured values were within the measured standard deviations. We hypothesize that, in practice, exceeding the typical hip angles by 1.2° should not substantially affect an active hip prosthesis user's stability or comfort. However, further testing with a prototype prosthesis is necessary to assess the effect of the hip angle algorithm on mobility.

Analysis of development and testing group data showed that hip max extension and hip max flexion angles during the gait cycle tended to vary slightly in a unique range for each person. Thus, each participant's hip max extension and flexion angles averaged over all strides were used as input constants for each participant in this study (Fig. 5). As a person walks, hip max extension and flexion angles remain consistent or vary slightly within a limited range, even with changes in walking speed. It is important to note that per-person unique constants were determined because people in both groups walked at speeds between 0.4 m/s to 1.5 m/s; therefore, an additional analysis could be required for people with preferred walking speeds outside this range. When the algorithm is used in practice for a powered hip control system, the clinician responsible for prosthetic fitting should adjust the hip max extension angle and hip max flexion angle constants to accommodate the user. These parameters would then be entered into the system as unique per-person constants.

Our feature and event-based approach has two key advantages over the echo controller approach proposed by Ueyama et al. [8]: our approach allows for gait speed adaptation, and also enables real-time control. These benefits are particularly valuable for prosthetic users who need to operate their prosthesis in environments where steady-state gait is not feasible. Echo controllers rely on the motion of the intact limb to determine joint trajectory and gait speed, which means it will take twice as long and require twice the distance to change gait speed [41]. By contrast, our proposed feature and event-based trajectory adjustment approach continuously adjusts the temporal and spatial aspects of hip kinematics in real-time, based on the user's response, which can be detected by moving the pelvis, applying foot-strike, and performing foot-off. This means prosthetic users can adjust their gait speed in real time without relying on the intact limb. Consequently, prosthetic users will be better equipped to navigate confined spaces or crowded environments where repeated and rapid speed adjustments are necessary. A desirable microprocessor-controlled prosthesis should seamlessly integrate all sensors within the prosthesis, allowing the user to simply charge and wear the device. However, most prostheses with echo controller integration are dependent on sensors located on the intact side and have not garnered positive feedback from users [9].

While we utilized correlation analysis to identify the relationship between pelvic motions and sagittal hip angles, the calculated hip rotation velocities based on these correlations were linear for all three periods (Fig. 1). Finite state impedance controller (virtual springs and dampers between thighs and pelvis) and virtual holonomic constraints can introduce angular velocity non-linearities that more closely resemble the biological kinematics [17, 34, 42]. Therefore, for future studies on active HKAF prosthesis control strategies, integration of finite state impedance controller or virtual holonomic constraints, in addition to our algorithm may be necessary to achieve appropriate hip kinematics. Additionally, bio signal analysis tools, such as wavelet analysis [43], and biomechanical pattern recognition approaches, such as machine learning and state estimation [44], could potentially be used to identify more pelvic motion features for a more accurate hip trajectory predication.

This research used gold-standard 3D motion capture data for pelvic kinematics measurements [45]. However, wearable systems such as lower limb prosthetics need integrated measurement technology to provide real-time input for prosthesis control. Recent studies have investigated the feasibility of using inertial measurement units (IMUs) to measure lower extremity kinematics [46, 47]. In a study by Berner et al. [46], multiple IMUs were used to measure the kinematics of different body segments with high accuracy. In another study, researchers accurately measured pelvic kinematics of an able-body participant by utilizing a single IMU mounted to the lower back [47]. In theory, the required pelvic motion input data for the developed algorithm could be provided by an IMU integrated into the socket connection component (i.e., IMU moves with the socket, which is attached to the pelvis, to provide pelvis kinematics) and a loadcell in the joint to measure applied forces such as at foot strike.

Considering the greater reliance of HD amputees on pelvic and trunk motions, further algorithm tuning of the pelvis feature detection and hip angle calculation algorithm are anticipated to optimize the prosthesis gait controller. By utilizing powered and intelligent hip joints, gait in people with an HD amputation could be improved to a level similar to people with transfemoral amputation. As powered hip prostheses become more widely available, new gait data could be collected with HD participants and used to improve the algorithm.

Ultimately, the algorithm proposed in this research is an important step towards creating a powered hip prosthesis controller that enables more efficient movement in the person's chosen environment, all with less strain on the lower back and less effort.

### Limitations

The testing group people walked on a treadmill at fixed speeds that were different than the development group. Furthermore, all gait cycle data used in this study were at a steady state. Therefore, it is possible that steady state walking reduced the natural gait variation and thus contributed to the high accuracy of our algorithm.

## Conclusion

A viable algorithm was developed and evaluated to calculate hip angles throughout the gait cycle using only pelvis angles and stance time data. This new hip angle calculation method lays the groundwork for the future development of an intelligent powered hip prosthesis control system since it permits the use of measurable pelvic data to generate hip data as user input for a person's HKAF prosthesis. Based on our gait kinematics and gait event time analysis, hip angle calculations would require sensors in the prosthesis capable of detecting pelvic kinematics and stance phase.

### Supplementary Information

**Additional file 1.** Real-time hip angle calculation algorithm

## Data Availability

The dataset acquired from Ottawa Hospital Research Institute is not authorized for external use. The dataset provided by S. Hood is available via scientific data database [26].

## Abbreviations

HD: Hip disarticulation
TF: Transfemoral
HKAF: Hip–Knee–Ankle–Foot
RMSE: Root-mean-square-error
Hθ_FS_: Hip angle at foot-strike instant
Hθ_E_: Maximum hip extension angle
Hθ_F_: Maximum hip flexion angle
Hτ_E_: Maximum hip extension time
Hτ_F_: Maximum hip flexion time
${{\overline{\text{H}\theta}}_{\text{E}}}$: Hip max extension angle constant
${{\overline{\text{H}\theta}}_{\text{F}}}$: Hip max flexion angle constant
PTθ: Pelvic tilt angle at foot strike
PR_ZC_: Timing of pelvic rotation angular velocity zero-crossing
ΔH: Hip rotation angle range of motion during pelvic rotation angular velocity zero-crossing
PT_ZC_: Pelvic tilt angular velocity first zero crossing
Τs: Stance time
PNO: Participant number
Corr: Pearson correlation

## References

[1] Fernández A, Formigo J (2005). Are Canadian prostheses used? A long-term experience. Prosthet Orthot Int.

[2] Chin T, Oyabu H, Maeda Y, Takase I, Machida K (2009). Energy consumption during prosthetic walking and wheelchair locomotion by elderly hip disarticulation amputees. Am J Phys Med Rehabil.

[3] Chin T, Kuroda R, Akisue T, Iguchi T, Kurosaka M (2012). Energy consumption during prosthetic walking and physical fitness in older hip disarticulation amputees. J Rehabi Res Dev..

[4] Ludwigs E, Bellmann M, Schmalz T, Blumentritt S (2010). Biomechanical differences between two exoprosthetic hip joint systems during level walking. Prosthet Orthot Int.

[5] Boone D (2020). Prosthetists and orthotists: an evolution from mechanic to clinician. Prosthet Orthot Int.

[6] Brandt A, Wen Y, Liu M, Stallings J, Huang HH (2017). Interactions between transfemoral amputees and a powered knee prosthesis during load carriage. Sci Rep.

[7] Martinez-villalpando EC, Herr H (2009). Agonist-antagonist active knee prosthesis: a preliminary study in level-ground walking. J Rehabil Res Dev.

[8] Ueyama Y, Kubo T, Shibata M (2019). Robotic hip-disarticulation prosthesis: evaluation of prosthetic gaits in a non-amputee individual. Adv Robot.

[9] Li L, Wang X, Meng Q, Chen C, Sun J, Yu H (2022). Intelligent knee prostheses: a systematic review of control strategies. J Bionic Eng.

[10] Varol HA, Goldfarb M. Real-time Intent Recognition for a Powered Knee and Ankle Transfemoral Prosthesis. IEEE.

[11] Ferreira C, Reis LP, Santos CP. Review of Control Strategies for Lower Limb Prostheses. Robot 2015: Second Iberian Robotics Conference. Advances in Intelligent Systems and Computing: Springer International Publishing; 2016. p. 209-20

[12] Tucker MR, Olivier J, Pagel A, Bleuler H, Bouri M, Lambercy O (2015). Control strategies for active lower extremity prosthetics and orthotics: a review. J Neuroeng Rehabil.

[13] Edelstein JE, Timothy LK, Ron S, John OB, Michael LM (2014). Amputations. Comprehensive guide to geriatric rehabilitation.

[14] Baum BS, Schnall BL, Tis JE, Lipton JS (2008). Correlation of residual limb length and gait parameters in amputees. Injury.

[15] Engstrom B, Van de Ven C (1999). Therapy for amputees.

[16] Devan H, Carman A, Hendrick P, Hale L, Ribeiro DC (2015). Spinal, pelvic, and hip movement asymmetries in people with lower-limb amputation: systematic review. J Rehabil Res Dev.

[17] Eslamy M, Oswald F, Schilling AF. Estimation of Knee Angles Based on Thigh Motion: A Functional Approach and Implications for High-Level Controlling of Active Prosthetic Knees. IEEE Control Systems: Institute of Electrical and Electronics Engineers Inc.; 2020. p. 49–61.

[18] Gailey RS, Roach KE, Applegate EB, Cho B, Cunniffe B, Licht S (2002). The amputee mobility predictor: an instrument to assess determinants of the lower-limb amputee's ability to ambulate. Arch Phys Med Rehabil.

[19] Wu G, Siegler S, Allard P, Kirtley C, Leardini A, Rosenbaum D (2002). ISB recommendation on definitions of joint coordinate system of various joints for the reporting of human joint motion–part I: ankle, hip, and spine. Int Soc Biomech J Biomech.

[20] Wu G, van der Helm FC, Veeger HE, Makhsous M, Van Roy P, Anglin C (2005). ISB recommendation on definitions of joint coordinate systems of various joints for the reporting of human joint motion–Part II: shoulder, elbow, wrist and hand. J Biomech.

[21] Sturk JA, Lemaire ED, Sinitski EH, Dudek NL, Besemann M, Hebert JS (2019). Maintaining stable transfemoral amputee gait on level, sloped and simulated uneven conditions in a virtual environment. Disabil Rehabil Assist Technol.

[22] Sinitski EH, Lemaire ED, Baddour N (2015). Evaluation of motion platform embedded with force plate-instrumented treadmill. J Rehabil Res Dev.

[23] Zeni JA, Richards JG, Higginson JS (2008). Two simple methods for determining gait events during treadmill and overground walking using kinematic data. Gait Posture.

[24] Sturk JA, Lemaire ED, Sinitski E, Dudek NL, Besemann M, Hebert JS (2018). Gait differences between K3 and K4 persons with transfemoral amputation across level and non-level walking conditions. Prosthet Orthot Int.

[25] Kendell C, Lemaire ED, Kofman J, Dudek N (2016). Gait adaptations of transfemoral prosthesis users across multiple walking tasks. Prosthet Orthot Int.

[26] Hood S, Ishmael MK, Gunnell A, Foreman KB, Lenzi T (2020). A kinematic and kinetic dataset of 18 above-knee amputees walking at various speeds. Sci Data..

[27] Hood S, Lenzi T. Lower Limb Kinetic and Kinematic Data of 18 Above Knee Amputees. 2020. 10.1038/s41597-020-0494-7 PMC7242470 32439980

[28] Vicon. Plug-in gait reference guide. In: Limited VMS, editor.2021.

[29] Collins TD, Ghoussayni SN, Ewins DJ, Kent JA (2009). A six degrees-of-freedom marker set for gait analysis: repeatability and comparison with a modified Helen Hayes set. Gait Posture.

[30] O'Sullivan K, Clifford A, Hughes L (2010). The reliability of the CODA motion analysis system for lumbar spine analysis: a pilot study. Physiother Pract Res.

[31] Eberly VJ, Mulroy SJ, Gronley JK, Perry J, Yule WJ, Burnfield JM (2014). Impact of a stance phase microprocessorcontrolled knee prosthesis on level walking in lower functioning individuals with a transfemoral amputation. Prosthet Orthot Int.

[32] Jaegers SM, Arendzen JH, de Jongh HJ (1995). Prosthetic gait of unilateral transfemoral amputees: a kinematic study. Arch Phys Med Rehabil.

[33] Shandiz MA, Farahmand F, Osman NAA, Zohoor H (2013). A robotic model of transfemoral amputee locomotion for design optimization of knee controllers. Int J Adv Robot Syst.

[34] Sup F, Bohara A, Goldfarb M (2008). Design and control of a powered transfemoral prosthesis. Int J Rob Res.

[35] Segal AD, Orendurff MS, Klute GK, McDowell ML, Pecoraro JA, Shofer J (2006). Kinematic and kinetic comparisons of transfemoral amputee gait using C-Leg and Mauch SNS prosthetic knees. J Rehabil Res Dev.

[36] Edelstein JE, Cameron MH, Monroe LG (2007). Amputations and prostheses. Physical rehabilitation.

[37] Koehler-McNicholas SR, Lipschutz RD, Gard SA (2016). The biomechanical response of persons with transfemoral amputation to variations in prosthetic knee alignment during level walking. J Rehabil Res Dev Serv..

[38] Heitzmann DWW, Leboucher J, Block J, Gunther M, Putz C, Gotze M (2020). The influence of hip muscle strength on gait in individuals with a unilateral transfemoral amputation. PLoS ONE.

[39] Blumentritt S, Ludwigs E, Bellmann M, Boiten H. The New Helix 3D Hip Joint. 2008.

[40] Sinitski EH, Lemaire ED, Baddour N, Besemann M, Dudek NL, Hebert JS (2015). Fixed and self-paced treadmill walking for able-bodied and transtibial amputees in a multi-terrain virtual environment. Gait Posture.

[41] Vallery H, Burgkart R, Hartmann C, Mitternacht J, Riener R, Buss M (2011). Complementary limb motion estimation for the control of active knee prostheses. Biomed Tech (Berl).

[42] Horn JC, Gregg RD (2022). Nonholonomic virtual constraints for control of powered prostheses across walking speeds. IEEE Trans Control Syst Technol.

[43] Akay M (1995). Wavelets in biomedical engineering. Ann Biomed Eng.

[44] Chalvatzaki G, Papageorgiou XS, Maragos P, Tzafestas CS. User-Adaptive Human-Robot Formation Control for an Intelligent Robotic Walker Using Augmented Human State Estimation and Pathological Gait Characterization. IEEE.

[45] Ferrari A, Benedetti MG, Pavan E, Frigo C, Bettinelli D, Rabuffetti M (2008). Quantitative comparison of five current protocols in gait analysis. Gait Posture.

[46] Berner K, Cockcroft J, Morris LD, Louw Q (2020). Concurrent validity and within-session reliability of gait kinematics measured using an inertial motion capture system with repeated calibration. J Bodyw Mov Ther.

[47] Bolink SA, Naisas H, Senden R, Essers H, Heyligers IC, Meijer K (2016). Validity of an inertial measurement unit to assess pelvic orientation angles during gait, sit-stand transfers and step-up transfers: Comparison with an optoelectronic motion capture system. Med Eng Phys.

