# Supplementary material for: A pelvic kinematic approach for calculating hip angles for active hip disarticulation prosthesis control
Source: J Neuroeng Rehabil. 2023 Nov 9;20:152. doi: 10.1186/s12984-023-01273-x (PMC10634065; doi:10.1186/s12984-023-01273-x)
Supplement: Supplementary file 1 — Additional file 1. Real-time hip angle calculation algorithm [file 12984_2023_1273_MOESM1_ESM.pdf]

Additional file 1

Real-time hip angle calculation algorithm

Sequence 1: Foot-strike to pelvic rotation zero-crossing time

$$CH\theta(t) = H\theta_{FS} + (P1\omega_{\alpha} \times t),\ 0 < t \leq PR_{ZC}$$

Where t is the gait time initiated at foot-strike (t=0 is foot-strike instance), CHθ(t) is the calculated hip angle throughout the gait time, $P1\omega_{\alpha}$ is the period 1 calculated constant angular velocity at foot-strike.

$$P1\omega_{\alpha} = \frac{\overline{H\theta}_E - H\theta_{FS}}{\acute{H}\tau_E - \tau_{FS}}$$

where $\overline{H\theta}_E$ is the per-person hip max extension angle constant, $H\theta_{FS}$ is the hip angle at foot-strike, $\acute{H}\tau_E$ is past-stride hip max extension time, and τ_{FS} is the foot-strike time. $\overline{H\theta}_E$ was determined for each participant based on their averaged hip max extension angle across all strides.

Sequence 2: pelvic rotation zero-crossing time to pelvic tilt zero-crossing time

$$CH\theta(t) = CH\theta(PR_{ZC}) + (P1\omega_{\beta} \times t),\ PR_{ZC1} < t \leq PT_{ZC}$$

Where $PR\tau_{ZC}$ is the pelvic rotation first zero-crossing time, PT_{ZC} is the pelvic tilt zero-crossing time, and $P1\omega_{\beta}$ is the calculated period 1 calculated constant angular velocity when PR_{ZC} is achieved.

$$P1_{\omega\beta} = 70 + 2.177 \times \left(\frac{\Delta H\theta}{\mathrm{PR}_{ZC} - \tau_{FS}} + P1\omega_{\alpha}\right) \div 2$$

where $P1\omega_{\beta}$ is the calculated constant angular velocity for sequence 2, $P1\omega_{\alpha}$ is the sequence 1 calculated constant angular velocity, τ_{FS} is the foot-strike time, PR_{ZC} is the pelvic rotation first zero-crossing time.

Sequence 3: pelvic tilt zero-crossing time to hip max extension time

$$CH\theta(t) = CH\theta(PT_{ZC}) + (P1\omega_{\beta} \times t), PT_{ZC1} < t \leq H\tau_E$$

Sequence 4: hip max extension time to stance time

$$CH\theta(t) = CH\theta(H\tau_E) + (P2\omega_{\alpha} \times t),\ H\tau_E < t \leq \tau_S$$

Where $P2\omega_{\alpha}$ is the calculated constant angular velocity at sequence 4, $H\tau_E$ is the calculated hip max extension time, τ_S is the foot-off time.

$$P2\omega_{\alpha} = \frac{\overline{H\theta}_F - CH\theta(H\tau_E)}{H\tau_{F\alpha} - H\tau_E}$$

where $\overline{H\theta}_F$ is the per-participant hip max flexion angle constant, $CH\theta(H\tau_E)$ is the calculated hip max extension angle at sequence 3, $H\tau_{MF\alpha}$ is the calculated hip max flexion time, and $H\tau_{ME}$ is the calculated hip max extension time.

Sequence 5: Stance time to hip max flexion time

$$CH\theta(t) = CH\theta(\tau_{FO}) + (P2\omega_{\beta} \times t),\ \tau_S < t \leq H\tau_{F\beta}$$

where $P2\omega_{\beta}$ is the calculated period 2 calculated constant angular velocity when τ_S is achieved, $H\tau_{F\beta}$ is the hip max flexion time, and τ_{FO} is the foot-off time.

Sequence 6: hip max flexion time to next foot-strike

$$CH\theta(t) = CH\theta(H\tau_{F\beta}),\ H\tau_{F\beta} < t$$
